# **γ**-H2AX: A Novel Prognostic Marker in a Prognosis Prediction Model of Patients with Early Operable Non-Small Cell Lung Cancer

**DOI:** 10.1155/2014/160236

**Published:** 2014-01-08

**Authors:** E. Chatzimichail, D. Matthaios, D. Bouros, P. Karakitsos, K. Romanidis, S. Kakolyris, G. Papashinopoulos, A. Rigas

**Affiliations:** ^1^Department of Electrical and Computer Engineering, Democritus University of Thrace, Xanthi, Greece; ^2^Department of Oncology, Democritus University of Thrace, Alexandroupolis, Greece; ^3^Department of Pneumonology, Democritus University of Thrace, Alexandroupolis, Greece; ^4^Department of Cytopathology, University of Athens Medical School, “Attikon” University Hospital, Athens, Greece; ^5^2nd Department of Surgery, Democritus University of Thrace, Alexandroupolis, Greece

## Abstract

Cancer is a leading cause of death worldwide and the prognostic evaluation of cancer patients is of great importance in medical care. The use of artificial neural networks in prediction problems is well established in human medical literature. The aim of the current study was to assess the prognostic value of a series of clinical and molecular variables with the addition of **γ**-H2AX—a new DNA damage response marker—for the prediction of prognosis in patients with early operable non-small cell lung cancer by comparing the **γ**-H2AX-based artificial network prediction model with the corresponding LR one. Two prognostic models of 96 patients with 27 input variables were constructed by using the parameter-increasing method in order to compare the predictive accuracy of neural network and logistic regression models. The quality of the models was evaluated by an independent validation data set of 11 patients. Neural networks outperformed logistic regression in predicting the patient's outcome according to the experimental results. To assess the importance of the two factors p53 and **γ**-H2AX, models without these two variables were also constructed. JR and accuracy of these models were lower than those of the models using all input variables, suggesting that these biological markers are very important for optimal performance of the models. This study indicates that neural networks may represent a potentially more useful decision support tool than conventional statistical methods for predicting the outcome of patients with non-small cell lung cancer and that some molecular markers, such as **γ**-H2AX, enhance their predictive ability.

## 1. Introduction

Prediction is one of the most interesting areas where intelligent systems are utilized [1]. In particular, prediction is an attempt to accurately forecast the evolution or outcome of a specific situation, using as input information a concrete set of variables that describe this situation [2]. In medicine, the valid and effective interpretation of medical data and the correct and early diagnosis along with a documented prognostic evaluation of the clinical and pathological data are very important parameters for a better management of the disease [3]. Prediction is a very difficult task because the expert human can hardly process the huge amount of data and usually suffers from absence of good and accurate analysis of these laboratory data [4, 5].

Lung cancer is the most common cause of cancer mortality worldwide for both men and women, causing approximately 1.2 million deaths per year. In the United States, there were 221.000 new cases of lung cancer and 157,000 deaths in 2011 [6]. The traditional evaluation of prognosis in non-small cell lung carcinoma (NSCLC) has relied, as in most other malignant tumors, on the stage of disease at the time of clinical presentation [7, 8]. Other factors currently commonly considered include performance status, weight loss, and presence or absence of symptoms at diagnosis, as well as time-honored pathologic parameters, for example, tumor size, tumor differentiation, and histological subtype [9, 10]. However, advances in molecular biology have provided important insights into other potentially significant prognostic biomarkers during the last decade such as the *γ*-H2AX histone [11]. Detection of *γ*-H2AX foci has been used as a biomarker for aging and cancer, as a biodosimeter for drug development and radiation exposure, as well as for clinical trials for cancer chemo- and radiotherapy [12–14].

The most popular choice for the prediction models in lung cancer is multivariable logistic regression (LR) model. In the report of Beane et al. [15] logistic regression models describe the likelihood of having lung cancer using the biomarker and clinical factors. Although in recent years, there has been growing interest in using artificial neural networks (ANNs) in order to predict lung cancer outcome, their accuracy has not been fully assessed. Consequently, there is a great need for formal evaluation and empirical comparison of neural networks with other conventional statistical methods. In the study of Santos-García et al. [16] an ensemble model of ANNs was proposed in order to predict the cardiorespiratory morbidity after pulmonary resection for non-small cell lung cancer. Despite the criticisms, such as greater computational burden and proneness to overfitting, supporters argue that neural networks provide more meaningful interpretations of data and conjecture that the performance of neural networks will surpass that of regression modelling techniques [17].

The aim of the present study was to predict the outcome of patients with early operable non-small cell lung cancer using ANNs by incorporating *γ*-H2AX, a new DNA damage response biomarker. The variables of the cohort included both clinical (sex, age, smoking status, TNM status, FEV1, history of adjuvant chemotherapy, and/or radiotherapy) and pathological (histological subtype, differentiation grade, Ki67, ploidy status, EGFR mutation status, apoptotic status (caspase 3), lymphatic and vascular infiltration). Additionally, the central role of two biological markers, p53 which is reported to be associated with pathogenesis of lung cancer and the histone *γ*-H2AX, has been investigated. This is the first study using *γ*-H2AX, a DNA damage biomarker, as input in a prognosis prediction model of patients with early operable non-small cell lung cancer.

## 2. Materials and Methods

Data from 96 patients with NSCLC disease were collected and recorded. All patients underwent radical thoracic surgery of primary tumour (lobectomy or pneumonectomy), together with regional lymph node excision between January 2002 and December 2005 at the Cardiac Surgery Department of Evangelismos Hospital. The corresponding data to each patient were structured in fields containing information about postsurgical measurements and type of treatment. Medical experts pointed out the importance of a set of prognostic factors selected from all the fields which were mentioned above. For each patient, 27 clinical markers were studied and are listed in Table 1. Histology reports were issued according to World Health Organization criteria. Staging was performed according to the 7th Edition of TNM in lung cancer. In addition, 11 of 96 patients who underwent surgery were used for validation of the models. The group comprised of 77 men and 19 women with ages ranging from 36 to 80 years (median age: 66, mean age ± SD: 65.64 ± 7.23 years) and included 42 adenocarcinomas, 42 squamous cell carcinomas, 8 large cell carcinomas, and 4 undifferentiated carcinomas. Two distinct classes of outcome are to be predicted: outcome class 0 refers to patient survival and outcome class 1 refers to death after the resection.

### 2.1. Artificial Neural Networks

ANNs are statistical models whose mathematical structure reproduces the biological organisation of neural cells for simulation of the learning dynamics of the brain [18]. In recent years, considerable attention has been paid to the application of ANN-based regression methods for the development of prognostic models in oncology [19]. Although some doubts have been raised about the real advantages of ANNs over traditional techniques [20], a recent review highlights their benefits for outcome prediction [21–23].

ANNs have been developed as generalizations of mathematical models of biological nervous systems [24–30]. A neural network has to be configured in such a way that the application of a set of inputs produces the desired set of outputs. Various methods to set the strengths of the connections exist. One way is to set the weights explicitly, using priori knowledge. Another way is to train the neural network by feeding it with teaching patterns and letting it change its weights according to some learning rule.

In order to implement an ANN, the network is processed in two levels, training and testing. In the level of training, the network is trained for an output prediction on the basis of input data. In the testing level, the network is used to predict an output. When the tested error reaches the desired tolerance value, the training of the network is stopped.

The back propagation (BP) algorithm is the most popular algorithm which has the widest area of use. The learning process in a BP network takes place in two steps. First, each pattern is presented to the network and propagated forward to the output. Second, a method called “gradient descent” is used to minimize the total error on the patterns in the training set. In gradient descent, the weights are changed in proportion with the negative of an error derivative with respect to each weight. The weights move in the direction of steepest descent on the error surface defined by the total error, where *N* is the number of patterns, *d*(*n*) is the predicted output, and *y*(*n*) the target:

(1) $$\begin{array}{c} \;\;\text{MSE}=\frac{1}{N}\overset{N}{\underset{n=1}{\sum }}{\left[d\left(n\right)-y\left(n\right)\right]}^{2}.\; \end{array}$$

The BP training algorithm is a gradient descent and its main function is to improve the performance of the network by reducing the total error through changing weights along its gradient. Finally, the tested mean squared errors (MSE) stop decreasing and they begin to increase, which is a sign of overtraining; the training is stopped.

### 2.2. Application of Artificial Neural Networks to Patient Data

The Neural Network Toolbox of Matlab was the software used for the artificial neural network development. Three independent models for patient estimation outcome at 1, 2, and 4 years after surgery were constructed. The output values of survival and death for each estimation model were set to 0 and 1, respectively. Multicollinearity would be expected to be present among some of the variables. However, calculation of the correlation coefficients for every combination of all variables in Table 1 revealed no disturbingly high correlation, and every correlation coefficient was found to be less than 0.85. Therefore, all 27 variables were used as potential inputs for the current analysis.

An MLP network which contains an input layer, a hidden layer, and an output layer was used. The number of units in the input layer is equal to the number of the input variables, while the output layer has only one unit which represents the status of the patient. After the ANN structure was designed, the data which were obtained in the experimental study were normalized in the 0-1 value set by using (2) in order to improve the characteristics of the training:

(2) $$\begin{array}{c} x_{\mathrm{norm}}=\frac{x-x_{\min}}{x_{\max}-x_{\min}}. \end{array}$$

Initially, the connection weight parameters were randomly assigned between 0 and 1, and subsequently they were automatically altered by the back propagation learning method to identify the optimal relationships between the input and the output. If useless variables are used as inputs of the ANN model, the accuracy of this model would be lower than that using only necessary variables. In addition to that, in cases where the number of connection weight parameters is much larger than that of the learning data set, the resultant model may have less generalizability and flexibility. Therefore, it was necessary to decrease the number of input and hidden units in order to optimize the model. For this reason, the parameter-increasing method (PIM) was used. The first step of PIM was to choose the most useful single input variable for accurate prediction. In the next step, the second most useful variable was selected. By repeating this operation, the best combination of input variables was selected in the prediction model. In the same way, the number of units in the hidden layer was decreased one by one from 10 in order to determine the ANN algorithm that yielded the best result.

The BP algorithm was used in the training procedure as well. Different transfer functions (purelin, tansig, logsig, etc.) were used and tried in the neurons in the hidden and output layers and logarithmic-sigmoid (logsig) was selected as the transfer function that yielded the best result.

The 5-fold cross-validation method was used for estimating the performance of the predictive model. At first, the 85 patients were divided into 5 subgroups. One of the 5 subgroups was used as evaluation data and the rest as learning data. The evaluation data were changed 5 times, such that each group was evaluated once as evaluation data. The average value of all accuracies of the evaluation data was considered as the estimation ability of the ANN model. In addition, a completely independent data set of 11 patients was also used to validate the ANN model constructed as described above.

The performance of the neural networks was estimated using judgement ratio (JR) and accuracy. Classification of a normal data as abnormal is considered as FP and classification of abnormal data as normal is considered as FN. TP and TN can be determined by the same way. The JR indicates the percentage of patients on which judgment can be achieved, while accuracy is used as a statistical measure of how well the binary classification correctly identifies or excludes the patient's outcome. JR and accuracy are presented in the following equations:

(3) $$\begin{array}{c} \text{JR}=\frac{N_{\text{TP}}+N_{\text{TN}}+N_{\text{FP}}+N_{\text{FN}}}{N_{\text{all}}}\times 100,\\ \text{Accuracy}=\frac{N_{\text{TP}}+N_{\text{TN}}}{N_{\text{TP}}+N_{\text{TN}}+N_{\text{FP}}+N_{\text{FN}}}\times 100, \end{array}$$

where *N*_TP_, *N*_TN_, *N*_FP_, *N*_FN_, and *N*_all_ are the number of TP, TN, FP, FN, and all collected data, respectively.

## 3. Results 

The logistic regression in this study was chosen as an accepted standard for prediction by biostatisticians in order to evaluate the modeling method [31]. SPSS for Windows (SPSS regression models 17.0, SPSS Inc., Chicago, IL) was used for LR modeling. The input variables for the LR model were optimized by PIM based on the likelihood ratio.

The selected variables for the 1-, 3-, and 4-year prediction models using ANN and LR in the order selected by PIM are shown in Table 2. The variables which are selected in the earlier steps are more useful for the l-year prediction. It was observed that several biological variables, especially p53 and ki67 as well as the histone *γ*-H2AX, were repeatedly selected in the models. The number of the units in hidden layers for the 1-, 3-, and 4-year prediction models was optimized resulting in 3, 5, and 2 units, respectively.

The JR and accuracy of the 1-, 3-, and 4-year prediction models using ANNs and LR are presented in Table 3. Both of them were much higher in the models using ANNs than those using LR in most of the cases. To assess the importance of the two factors, p53 and *γ*-H2AX, models without these two variables were also constructed. JR and accuracy of these models were lower than those of the models using all input variables (Tables 4 and 5), suggesting that these biological markers are very important for optimal performance of the models.

In order to investigate the efficacy of the constructed ANN model, an independent data set of 11 patients, who underwent surgery, was used for validation. JR was 72.7% and 81.8% for 3- and 4-year prediction of the outcome, respectively (Table 6).

## 4. Discussion

With the development of ANNs as an alternative method to logistic regression for prediction, research has been conducted to investigate the differences between the two techniques [32, 33]. There are many advantages and disadvantages to the use of artificial neural networks as a classification tool. ANNs have an excellent capability of learning the relationship between the input-output mapping from a given dataset without any prior knowledge or assumptions about the statistical distribution of the data. This capability of learning from a certain dataset without any priori knowledge makes the neural networks quite suitable for classification and prediction tasks in practical situations. Furthermore, neural networks are inherently nonlinear which makes them more practicable for accurate modelling of complex data patterns, as opposed to many traditional methods based on linear techniques. Due to their behaviour, they have found application in a wide range of medical fields such as cardiology, gastroenterology, pulmonology, oncology, neurology, and paediatrics [20–27].

One of the disadvantages of ANNs when compared to logistic regression models is that ANNs frequently have difficulty analyzing systems which have a large number of inputs due to the large amount of time taken to learn the system as well as possibly overfitting the model during the learning time. Linear and logistic regression models have less potential for overfitting primarily because the range of functions they can model is limited.

Recently the task of comparison between these two models has been addressed from different points of view. Several published works in the medical literature have demonstrated the success of the ANN approaches. In a review carried out by Sargent et al. on 28 major studies, ANN outperformed logistic regression in 10 cases (36%) and was outperformed by regression in 4 cases (14%) and the 2 methods had similar performance in the remaining cases. Sargent concluded that both methods should continue to be used and explored in a complementary manner [34].

In this study, ANNs and LR achieved promising prediction results when clinical parameters and molecular factors were considered simultaneously in the prediction model. The predictive ability of ANNs was found to be comparable to that of the logistic regression model. Specifically, the ANN models significantly outperformed logistic models in terms of accuracy. ANNs had a prediction success rate of about 88%. Although the success rate of correct prediction was not 100%, this study shows that the rate can be improved step by step when parameters and novel molecular parameters involved in lung cancer are added and considered together.

Moreover, the present study was able to show each factor's importance priority in lung cancer. For the first time, *γ*-H2AX, a DNA damage biomarker, was used in a prognosis prediction model of patients with early operable non-small cell lung cancer. Our research team was the first to demonstrate that overexpression of *γ*-H2AX may represent an independent prognostic indicator of worse overall survival in patients with non-small cell lung cancer [35].

## 5. Conclusions

In conclusion, our study demonstrated that the incorporation of *γ*-H2AX in an artificial network prediction model for patients with early operable NSCLC outperformed logistic models in terms of accuracy. A better prediction of non-small cell lung cancer prognosis will be possible by increasing patient's data, adding appropriate input parameters as biomarkers, and using artificial intelligence methods that can work together with ANNs.

## Figures and Tables

**Table 1:** Characteristics of the prediction variables.

Variables	No. of patients	Percentage (%)
Total number of patients	96	
Age (years)		
Mean ± SD	65.64 ± 7.23	
<65	36	37.5
≥65	60	62.5
Gender		
Male	77	80.2
Female	19	19.8
Histology		
Adenocarcinoma	42	43.8
Squamous cell carcinoma	42	43.8
Large cell carcinoma	8	8.3
Undifferentiated carcinoma	4	4.1
T status		
T1	13	13.5
T2	65	67.7
T3	18	18.8
Regional lymph node status (N)		
N0	50	52.1
N1	27	28.1
N2	19	19.8
Stage		
I	41	42.7
II	22	22.9
III	31	32.3
IV	2	2.1
Grade		
Low	25	26
Medium/high	71	74
Border of bronchus		
Positive	12	12.5
Negative	84	87.5

FEV1 < 70%	17	17.7
Positive vessel infiltration	41	42.7
Positive lymphatic infiltration	21	21.9
Positive pleural infiltration	46	47.9
Chemotherapy adjuvant	72	75.0
Radiotherapy adjuvant	41	42.7
High expression of P53	55	57.3
High expression of caspase 3	24	25.0
High expression of *γ*-H2AX	25	26.0
High expression of Ki67	29	30.2

**Table 2:** Selected input variables by the parameter-increasing method.

Order of selection	1-year survival		3-year survival		4-year survival	
	ANN	LR	ANN	LR	ANN	LR
1	High expression of *γ*-H2AX	High expression of *γ*-H2AX	High expression of Ki67	High expression of Ki67	Age	N2 number
2	High expression of caspase 3	Chemotherapy adjuvant	High expression of caspase 3		Histology	Histology
3	First metastasis	Radiotherapy adjuvant	N1 number		Grade	Border of bronchus
4	High expression of P53		Grade		N	
5	Gender		High expression of P53		Radiotherapy adjuvant	
6					High expression of caspase 3	
7					High expression of *γ*-H2AX	
8					Fev1 < 70%	
9					High expression of P53	

**Table 3:** Comparison of predictive models for 1-, 3-, and 4-year using ANN and LR.

	1-year prediction		3-year prediction		4-year prediction	
	ANN	LR	ANN	LR	ANN	LR
JR (%)	87.1	88.2	88.2	87.1	88.2	87.1
No. of patients	74/85	75/85	75/85	74/85	75/85	74/85
Accuracy (%)	93.3	90.7	78.7	74.3	92	91.9
No. of patients	69/74	68/75	59/75	55/74	69/75	68/74

**Table 4:** Selected input variables by the parameter-increasing method.

Order of selection	1-year prediction	1-year prediction without variables p53 and ph2ax
1	High expression of *γ*-H2AX	High expression of caspase 3
2	High expression of caspase 3	Stage
3	First metastasis site	First metastasis site
4	High expression of P53	Border of bronchus
5	Gender	Fev1 < 70%

**Table 5:** Comparison of predictive models for 1-year prediction.

	1-year prediction	1-year prediction without variables p53 and *γ*-h2ax
JR (%)	87.1	72.3
No. of patients	74/85	62/85
Accuracy	93.3	85.4
No. of patients	69/74	53/62

**Table 6:** Estimation of 3- and 4-year outcome prediction for unlearned data set.

	3-year prediction		4-year prediction	
	Learning data	Validation data	Learning data	Validation data
JR (%)	88.2	72.7	88.2	81.8
No. of patients	75/85	8/11	75/85	9/11
Accuracy (%)	78.7	75	92	88.9
No. of patients	59/75	6/8	69/75	8/9
